# Taxonomic and functional alterations in the salivary microbiota of children with and without severe early childhood caries (S-ECC) at the age of 3

**DOI:** 10.7717/peerj.13529

**Published:** 2022-05-31

**Authors:** Zhe Tang, Wenyi Xu, Zhifang Zhou, Yanchun Qiao, Shuguo Zheng, Wensheng Rong

**Affiliations:** 1 Department of Preventive Dentistry, Peking University School and Hospital of Stomatology, Beijing, China; 2 Beijing QuantiHealth Technology Co., Ltd., Beijing QuantiHealth Technology Co., Ltd., Beijing, China; 3 Department of Stomatology, Beijing Shijitan Hospital, Capital Medical University, Beijing, China

**Keywords:** Severe early childhood caries (S-ECC), Shotgun sequencing, KEGG analysis, Carbohydrate metabolism, Salivary microbiota

## Abstract

**Background:**

Primary dental caries is the most prevalent oral disease among preschool children, which can cause severe damage to teeth and even affect the mental well-being of children. Various studies have demonstrated that the oral microbiome plays a pivotal role in the onset and development of dental caries. However, it remains uncertain about the key microbial markers associated with caries, owing to the limited evidence.

**Methods:**

Fifteen S-ECC children and fifteen healthy controls were selected from three-year-old children in this study. Their clinical data and oral saliva samples were collected. Shotgun sequencing was conducted to investigate the microbial differences and the relevant functions between the two groups.

**Results:**

We observed no apparent difference in oral microbial community diversity between the two groups. Still, at the genus/species levels, several characteristic genera/species such as *Propionibacterium*, *Propionibacterium acidifaciens, Prevotella denticola, Streptococcus mutans* and *Actinomyces sp. oral taxon 448/414* increased significantly in S-ECC children, compared with the oral health group. Furthermore, we found that functional pathways involving glycolysis and acid production, such as starch and sucrose metabolism, fructose and mannose metabolism, glycolysis/gluconeogenesis, were prominently up-regulated in the high-caries group.

**Conclusions:**

Our study showed that dental caries in children were associated with the alterations in the oral microbiota at the composition and functional levels, which may potentially inspire the exploration of microbial diagnosis or therapeutic treatments.

## Introduction

According to the Global Burden of Disease (GBD) study in 2017, 3.5 billion people worldwide have suffered from oral diseases, and 532 million children had untreated primary dental caries (GBD 2017 Oral Disorders Collaborators, 2020). Caries is one of the most common oral problems among children. The 4th National Oral Health Survey in China reported that the caries prevalence rates of children aged 3, 4 and 5 were 50.8%, 63.6%, and 71.9%, respectively, with the corresponding decayed-missing-filled tooth (dmft) values of 2.28, 3.40, and 4.24. Du et al. (2018) recently displayed that the prevalence and degree of caries in preschool children in China were increasing with age and more than 70% of 5-year-old children have primary dental caries. Primary dental caries is prone to destruct hard tooth tissues and cause toothache, thus interfering with children’s daily lives. Importantly, the tooth loss occurring in serious cases may bring adverse impacts on children’s growth and their mental health (Hemadi et al., 2017). Therefore, it is necessary to perform the early diagnosis and effective interventions to primary dental caries.

For the etiology of dental caries, bacteria play vital roles in the onset and progression, as described in the four-factor theory of dental caries (Newbrun, 1989). Currently, Marsh’s “ecological plaque theory” is the well-recognized one of caries etiology (Marsh, 1994). In this theory, the low cariogenic bacteria in the original steady-state can turn to be high cariogenic bacteria driven by the frequent intake of fermentable carbohydrates. The produced organic acids consequently increase, thereby leading to the demineralization of tooth hard tissues and ultimately dental caries. Hence, oral bacteria, namely the oral microbiome, are emerging as intriguing spots for researchers to emphasize.

In recent decades, the high-throughput sequencing methodology has been rapidly developed and improved, facilitating researchers to study the crosstalk between the oral microbiome and host health (Zhou et al., 2018; Wu et al., 2018). Such studies not only enrich our understanding of the involvement of microorganisms in oral diseases, but also provide a novel microbial basis for the clinical diagnosis and potential therapeutic treatments. For instance, Huang et al. (2014) have reported a predictive modeling of gingivitis severity and susceptibility *via* oral microbiota, containing two indices: microbial index of gingivitis (MiG) and MiG-sensitivity index, by which patients could be accurately classified according to the severity and sensitivity to gingivitis. Besides, a new antibiotic named specifically targeted anti-microbial peptide (STAMP) was once proposed by Eckert et al. (2006). STAMP has been claimed to selectively target and kill caries-causing *streptococcus mutans* and reverse the oral microecological imbalance caused by the overgrowth of *streptococcus mutans*, thus achieving precise control of caries and ecological prevention (Eckert et al., 2006; Guo et al., 2015). With respect to the microbiome studies of caries, investigations on dental plaque using the denaturing gradient gel electrophoresis (DGGE) and 16S rDNA sequencing have revealed that the microbial diversity of severe early childhood caries (S-ECC) group was lower than that of caries-free group (Li et al., 2007; Zhang et al., 2020a; Zhang et al., 2020b). Another team conducted 16S rRNA sequencing analysis of unstimulated salivary microorganisms collected from children with and without caries (Jiang et al., 2016). They reported no significant differences in salivary microbiota diversity between the two groups, exhibiting inconsistent results with the two studies mentioned above. It is plausible that different samples and sequencing/analytical approaches may affect the readouts. To gain a more accurate and comprehensive illustration, metagenome shotgun sequencing has been introduced into the oral microbiome studies. One research in 2012, for the first time, applied shotgun sequencing to compare the oral microbiota between oral health adults and population with oral cavity (Belda-Ferre et al., 2012). They reported that the imbalance of the oral microbial community was tightly connected with the occurrence of caries. However, bacteria markers specific for caries still could not be identified in that work. In light of the limited microbial data related to dental caries in children and the rapid development of the microbiome field, more in-depth and systematic researches are urgently required to explore the relationship between primary dental caries and microbial flora structure and functional metabolisms.

In the present study, we collected saliva samples from 15 S-ECC (severe early childhood caries) preschool children and 15 caries-free children with the same ages (36∼42 months). Shotgun sequencing was used to analyze the influence of caries on the structure, composition, and functions of the oral microbiome. Although the diversities of microbial communities were not obviously altered in caries children, specific genus/species (*e.g.*, *Propionibacterium*, *Propionibacterium acidifaciens*, *Prevotella denticola*, *Streptococcus mutans* and *Actinomyces sp. oral taxon 448/414*) were significantly up-regulated in the S-ECC group, as well as several functional pathways involving carbohydrate metabolism. Hence, our work demonstrated that prominent differences in salivary microbiota existed between S-ECC children and caries-free children by shotgun sequencing, providing a microbial basis for researchers to comprehensively understand the pathogenesis and progression of early childhood caries.

## Materials & Methods

### Study subjects

The study was approved by the Ethics Committee of Peking University School and the Hospital of Stomatology (PKUSSIRB-201736081) and agreements were signed by all subjects’ parents before being registered.

Subjects in the study were preschool children recruited from the same kindergarten in Shijingshan District, Beijing, China. Oral examinations were performed for all subjects during the first visit by the same dentist. The oral standards for caries referred to the WHO oral health survey basic methods (the 5th edition). Briefly, the diagnosis of severe early childhood caries (S-ECC) consists of three major parts: (1) any sign of smooth-surface caries in a child younger than three years old, (2) the presence of one or more decayed, missing (due to caries), or filled smooth surfaces in any primary maxillary anterior teeth in children aged from three to five, (3) dmft (decayed, missing, filled tooth) index ≥ 4 (age 3), or dmft ≥ 5 (age 4), or dmft ≥ 6 (age 5) (American Academy of Pediatric Dentistry & American Academy of Pediatrics, 2016). S-ECC would be diagnosed if any of these criteria is satisfied.

All selected subjects should meet these following criteria: 3–3.5 years old (36–42 months), no less than 18 teeth, no orthodontic appliances and accessories, no use of antibiotics, probiotics, synbiotics or fluoridated toothpaste and microecological regulators within the recent three months, no apparent active bacteria or viral infection in any parts of the body, no systemic diseases or congenital disease (Xu et al., 2018). S-ECC group contained 15 children with dmfs (decayed, missing, filled surface) ≥ 8. Oral healthy group had 15 children with dmfs = 0 and no treatment on teeth. Age and gender of the healthy group matched with the S-ECC group.

### Sample collection

Oral saliva was collected according to the NIH human microbiology program (https://commonfund.nih.gov/hmp). The sampling process was carried out in the morning on the same day, without brushing teeth the night before and without eating foods for at least two hours before sampling. Participants were asked to open their mouths and stop swallowing to allow saliva to flow into a sterile cryogenic vial with the preservation buffer, which were provided by Beijing QuantiHealth Technology Co., Ltd (Beijing, China). A 2-ml sample of spontaneous, non-stimulated whole saliva was obtained from each individual. Samples were then labeled and immediately frozen in dry ice. After sampling, all salivary samples were transferred to Beijing QuantiHealth Technology Co., Ltd. The collected samples were stored at −80 °C before DNA extraction.

### Microbial DNA extraction

Salivary samples were sent to Beijing QuantiHealth Technology Co., Ltd. (Beijing, China) for DNA extraction and sequencing. Oral genomic DNA Extraction Kit (Takegene^®^, Ningbo ZD Biotechnology Co., Ltd.) was used to extract and purify DNA. The extracted DNA was evaluated by 1% agarose gel electrophoresis. DNA concentration and quality were determined with NanoDrop 2000UV—vis spectrophotometer (Thermo Fisher Scientific, Waltham, MA) and Qubit3.0 fluorometer (Thermo Fisher Scientific, Waltham, MA).

### Shotgun sequencing and quality control

DNA library was prepared using the KAPA HyperPlus Library Preparation Kit and KAPA Library Quantification Kit (KAPA Biosystems), following the manufacturer’s instructions. Shotgun sequencing was performed on an Illumina Novaseq 6000 System (Illumina).

All raw sequencing data were quality-controlled by MOCAT2 software (Kultima et al., 2016). Specifically, the Cutadapt software (v1.14, parameter: - M 30) was used to remove sequencing adapters (Martin, 2011). SolexaQA package was used to remove the reads with a threshold of less than 20 or a length of less than 30bp (Cox, Peterson & Biggs, 2010). The filtered reads were then compared with the host genome using SOAPaligner (v2.21, parameter: - M 4 - L 30 - V 10) to remove the contamination of host reads, and thus high-quality clean data were obtained (Li et al., 2009). The clean reads were assembled by SOAP de novo software (v2.04, parameter: all-D 1-m 3-L 500), and the scaftigs with a length of larger than 500 bp were obtained. Genes were predicted using MetaGeneMark (Zhu, Lomsadze & Borodovsky, 2010). A non-redundant gene catalog was constructed with CD-HT Redundancy was removed using CD-HIT.

### Data processing

The relative abundance of bacteria was obtained from the phylum to species levels by comparing clean reads with clade-specific markers using MetaPhlAn3. Based on the taxonomy information, α-diversity (including indices of Shannon, Simpson, Richness, and Evenness) was evaluated using the diversity function of R package vegan. PCoA analysis was performed based on the Bray-Curtis distance. LEfSe was used to detect the species with significant abundance differences between two groups according to the nonparametric factor Kruskal-Wallis rank-sum test, after analyzing the relative abundance data based on MetaPhlAn3. Wilcoxon rank-sum test of groups was used to analyze the differences between the two groups. Linear discriminant analysis (LDA) was used to reduce the dimension of the data and evaluate the influence of species with significant differences (LDA score). For functional analysis, the clean reads were processed with HUMAnN2 (http://huttenhower.sph.harvard.edu/humann2) to obtain the relative abundance of KO (KEGG Orthology). The profiles of KEGG levels were generated based on KO abundance and the KEGG database.

### Statistical analysis

SPSS software (SPSS version 26) was used to compare subjects. Two-tailed independent-samples *t*-tests was used to analyze the demographic and clinical data we obtained. To evaluate microbial diversity, the difference between two groups was calculated by PREMANOVA. The nonparametric Wilcoxon test was performed to evaluate differences in the relative abundance of metagenomics features.

## Results

### Sample collection and sequencing features

In the present study, 30 preschool children aged three-year old were recruited following the experimental designs and were equally divided into two groups: the S-ECC group (indicated as caries group in figures) and the caries-free group (indicated as healthy group in figures). The age and gender ratio of participants matched between two groups and the demographic and clinical information of all participants were listed in Table 1. Non-stimulated saliva samples were collected from children for the following oral microbiota investigation.

**Table 1:** Demographic and clinical data of children with S-ECC and healthy controls.

Characteristics	Children with S-ECC (*n* = 15)	Healthy controls (*n* = 15)	*P* value
Gender (M/F)	8/7	8/7	
Age (Mean ± SD, months)	42.25 ± 1.70	42.18 ± 2.30	0.480
dmft^**^(Mean ± SD)	11.40 ± 2.35	0 ± 0	<0.01
dmfs^**^(Mean ± SD)	24.67 ± 10.51	0 ± 0	<0.01

**Notes.**

Two asterisks (**) *P* < 0.01.

We carried out shotgun sequencing at a depth of 1 GB using the qualified microbial DNAs. 192.51 GB raw data of 30 samples were obtained, with an average of 6.42 GB raw data in each sample. After quality control, 81.71 GB data were obtained, with an average of 2.72 GB clean reads for each sample. 737,636,765 sequences in total were generated, including: 279,805,186 effective sequences from 15 subjects in healthy group (with 18,653,679 sequences in each sample) and 457,831,579 effective sequences from 15 subjects in caries group (with 30,522,105 sequences in each sample). Furthermore, we identified six phyla, 62 genera and 231 species, and 2,420,045 genes through the taxonomic annotation and gene assembly.

### Microbial diversity analysis of oral microbiome

**Figure 1:**
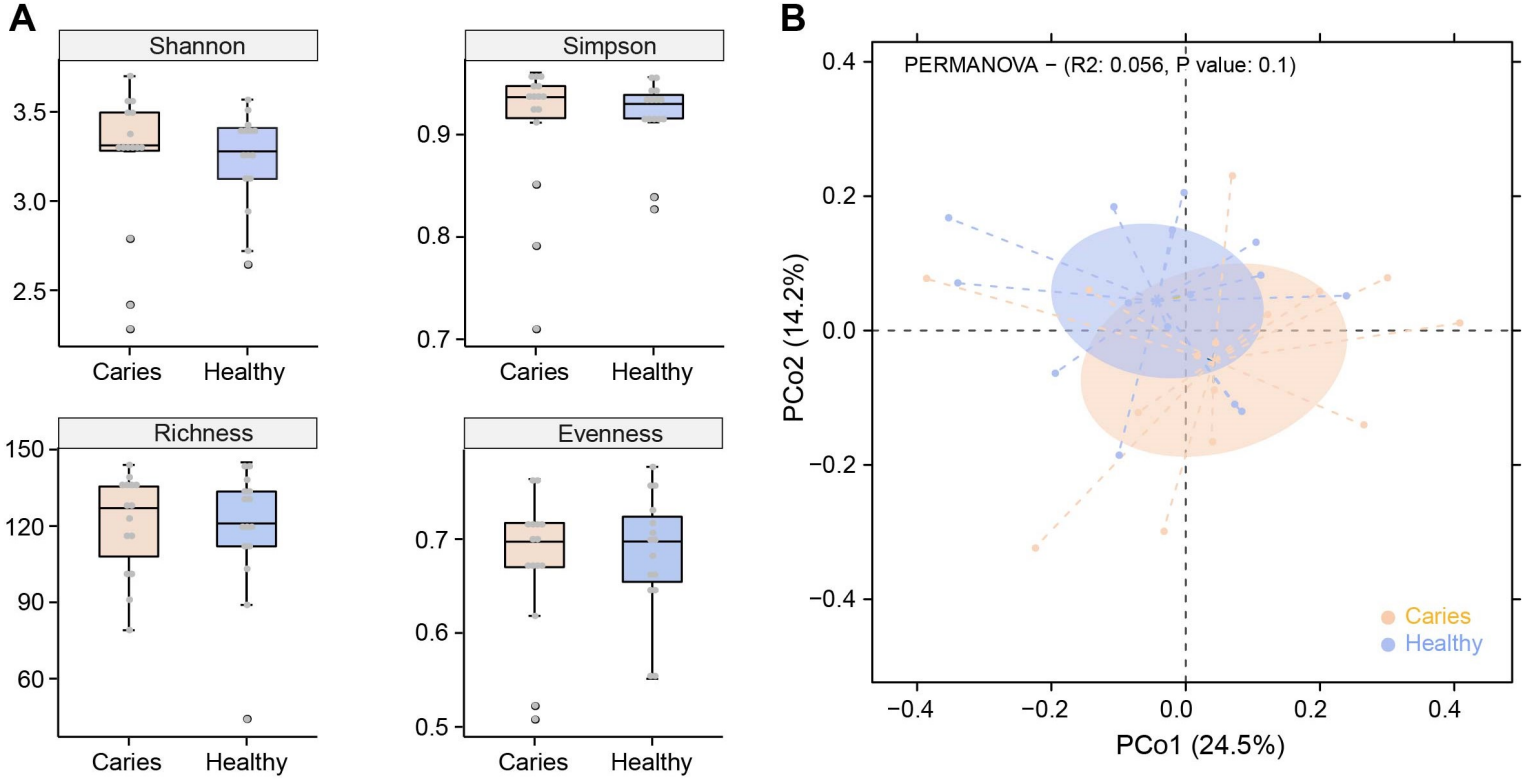
Comparisons of microbial diversities between S-ECC children (Caries) and healthy controls (Healthy). Alpha diversity was evaluated by indices of Shannon, Simpson, richness and evenness (A). (B) Comparison of principal coordinates analysis (PCoA) of two groups. Each dot represents one sample.

Due to the different sequencing samples and technologies, there is no assured conclusion regarding the influence of caries on the oral microbiota in children. In this study, we first performed α-diversity analysis to compare indices values of Shannon, Simpson, richness and evenness between S-ECC and healthy groups. As shown in Fig. 1A, no obvious difference in α-diversity was observed between the two groups. Similarly, samples in caries and caries-free groups were not separated according to the subsequent PCoA (principal co-ordinates analysis) analysis (Fig. 1B). We found a slight shift in two microbial communities, yet with no significant difference. Our results suggested that caries status barely interrupted the community structures of salivary microbiota in preschool children.

### Regulation of caries on oral microbial composition

**Figure 2:**
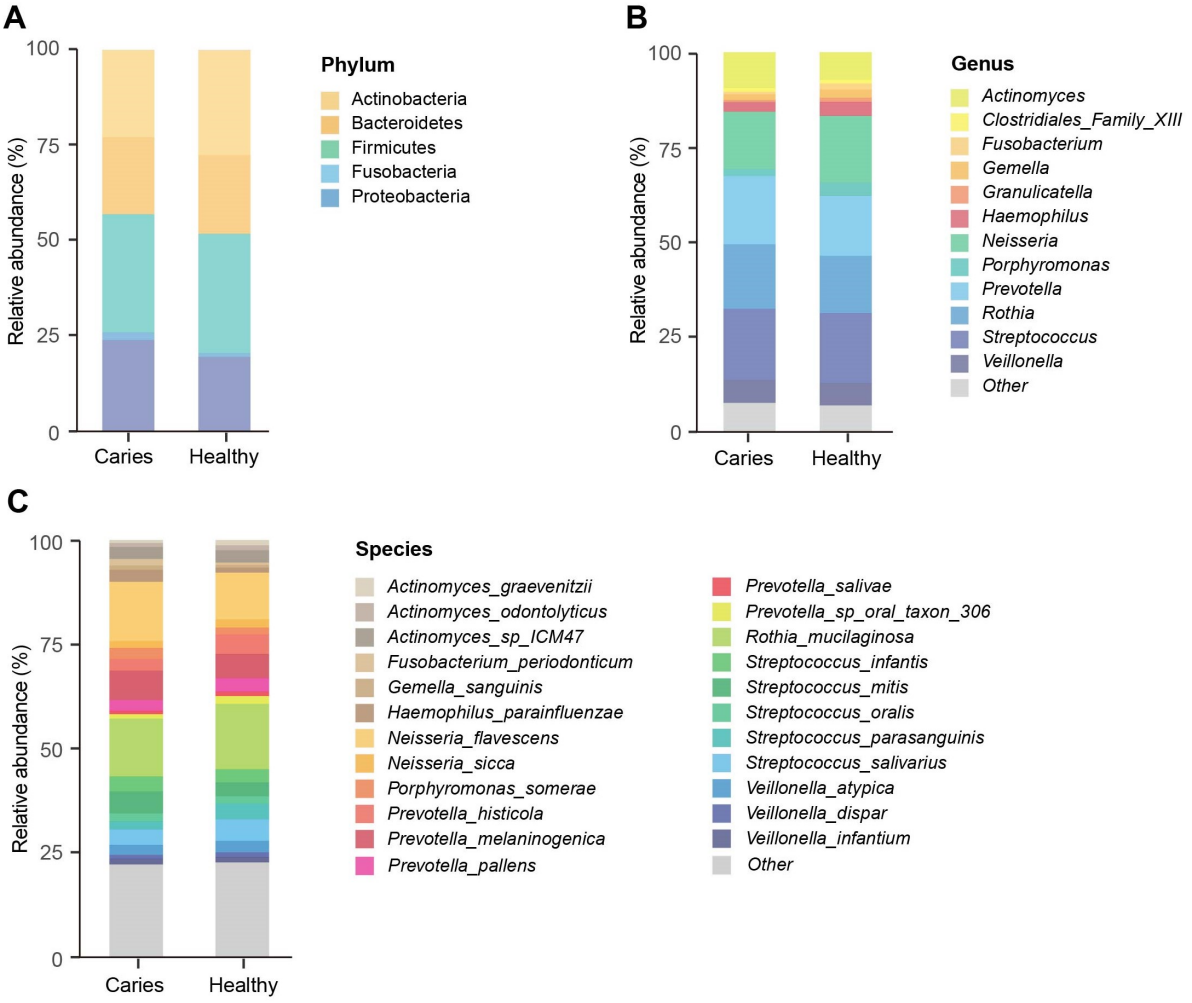
The microbial composition analysis of S-ECC and healthy children at the phylum/ (A) genus (B) species (C) levels.

We next investigated whether the composition of the oral microbiota was altered by the occurrence of dental caries. Five dominant phyla in caries and healthy groups were identified, including Firmicutes, Actinobacteria, Proteobacteria, Bacteroides and Fusobacteria, accounting for 99.99% of the annotated phyla (Fig. 2A). Among them, the relative proportion of Proteobacteria in the S-ECC group was higher than that of caries-free group, while Actinobacteria was down-regulated in the S-ECC group, as compared with oral healthy children, indicating that dental caries significantly affected Proteobacteria and Actinobacteria. At the genus level, we identified and ranked the top 12 genera by the average relative abundance: *Actinomyces, Clostridiales Family XIII, Fusobacterium, Gemella, Granulicatella, Haemophilus, Neisseria, Porphyromonas, Prevotella, Rothia, Streptococcus* and *Veillonella* (Fig. 2B). Compared with the caries-free group, both *Neisseria* and *Haemophilus* were elevated in the S-ECC group, and the relative level of *Actinomyces* significantly decreased. Given the superiority of shotgun sequencing, we further carried out the taxonomic annotation down to the species level and found several species were differentially distributed in caries and healthy children. For instance, the relative abundance of *Neisseria flavescens* and *Haemophilus parainfluenzae* were prominently greater in the S-ECC group, and the proportion of *Actinomyces graevenitzii* was lower than that in healthy group (Fig. 2C).

**Figure 3:**
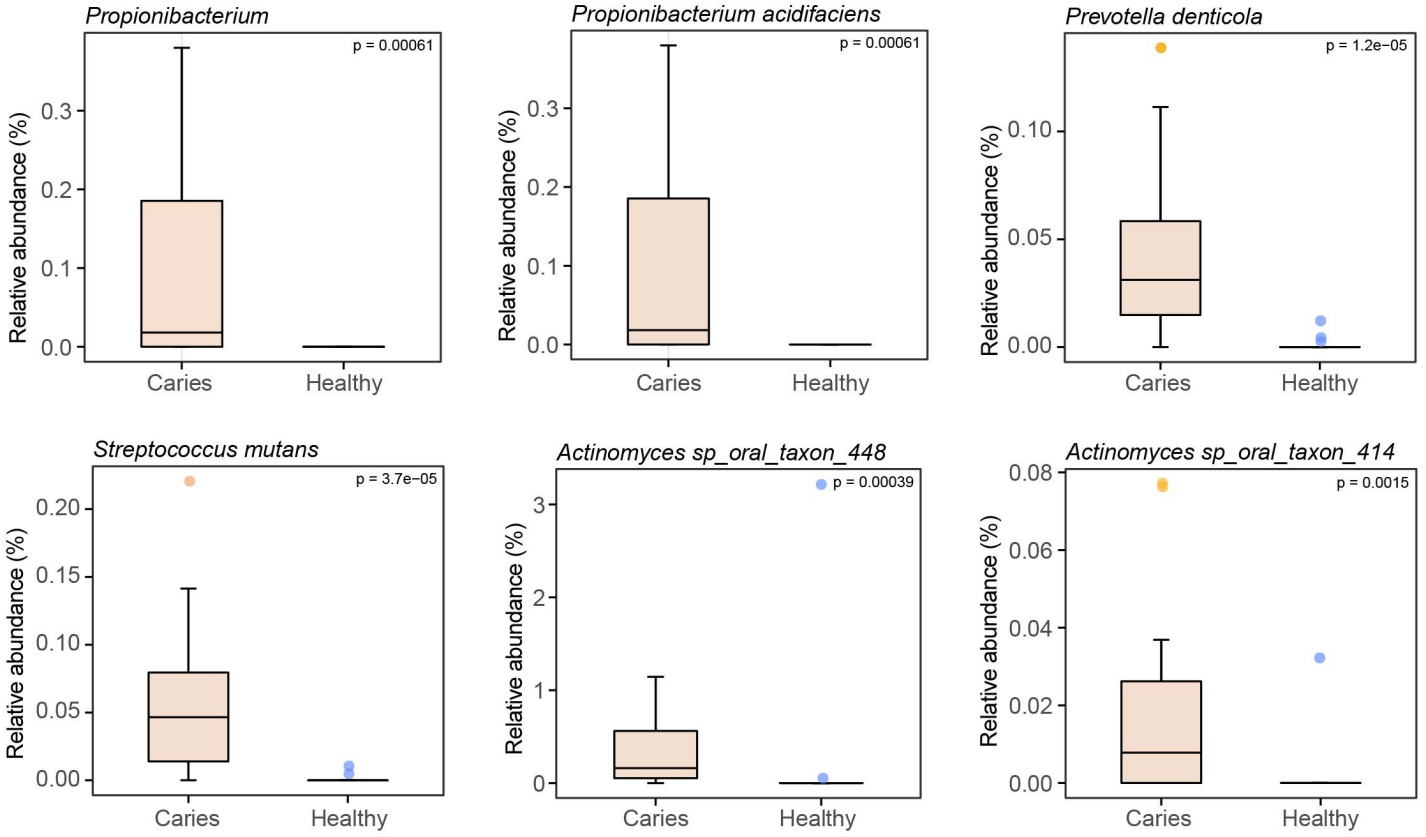
Relative abundances of several key genus/species.

**Figure 4:**
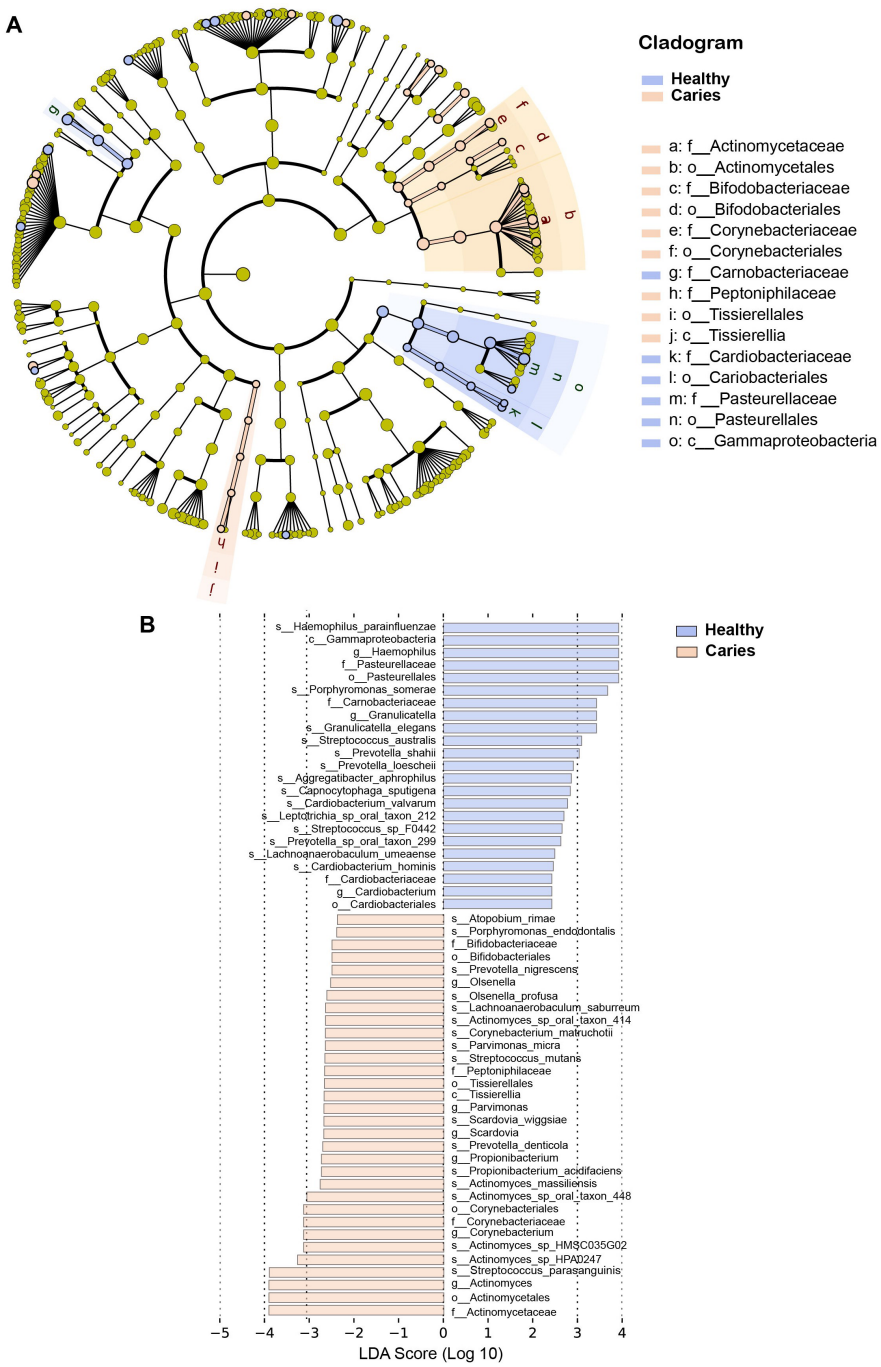
(A-B) LEfSe analysis between S-ECC children and caries-free children. The threshold of LDA score was two.

By Wilcoxon rank-sum permutation test, we identified a series of characteristic genus/species, including *Propionibacterium, Propionibacterium acidifaciens, Prevotella denticola, Streptococcus mutans* and *Actinomyces sp. oral taxon 448/414*, whose relative levels were significantly up-regulated in the caries group (Fig. 3). More importantly, *Propionibacterium* and *Propionibacterium acidifaciens* were absent in caries-free children, but it was detected in the salivary microbiota of S-ECC children. These findings indicated that these species could be the potential characteristic bacteria associated with the occurrence of primary dental caries. Additionally, we performed LEfSe (LDA effect size) analysis to explore the specific genera/species between S-ECC group and caries-free group, aiming to find out possible biomarkers between the two groups. As shown in Fig. 4, the enriched bacteria in children with and without caries were significantly distinct (using an LDA score threshold of 2). Also, the aforementioned typical genus/species (*Propionibacterium, Propionibacterium acidifaciens, Prevotella denticola, Streptococcus mutans* and *Actinomyces sp. oral taxon 448/414*) were found to increase in the S-ECC group, which was highly consistent with our previous analysis.

Collectively, our results showed that the oral microbiome, to be precise, the composition of salivary microbiota was altered in preschool children with dental caries, compared to oral health children, and several indicated genus/species might be microbial markers connecting with primary dental caries.

### Functional profiles of the oral microbiota in caries and healthy subjects

**Figure 5:**
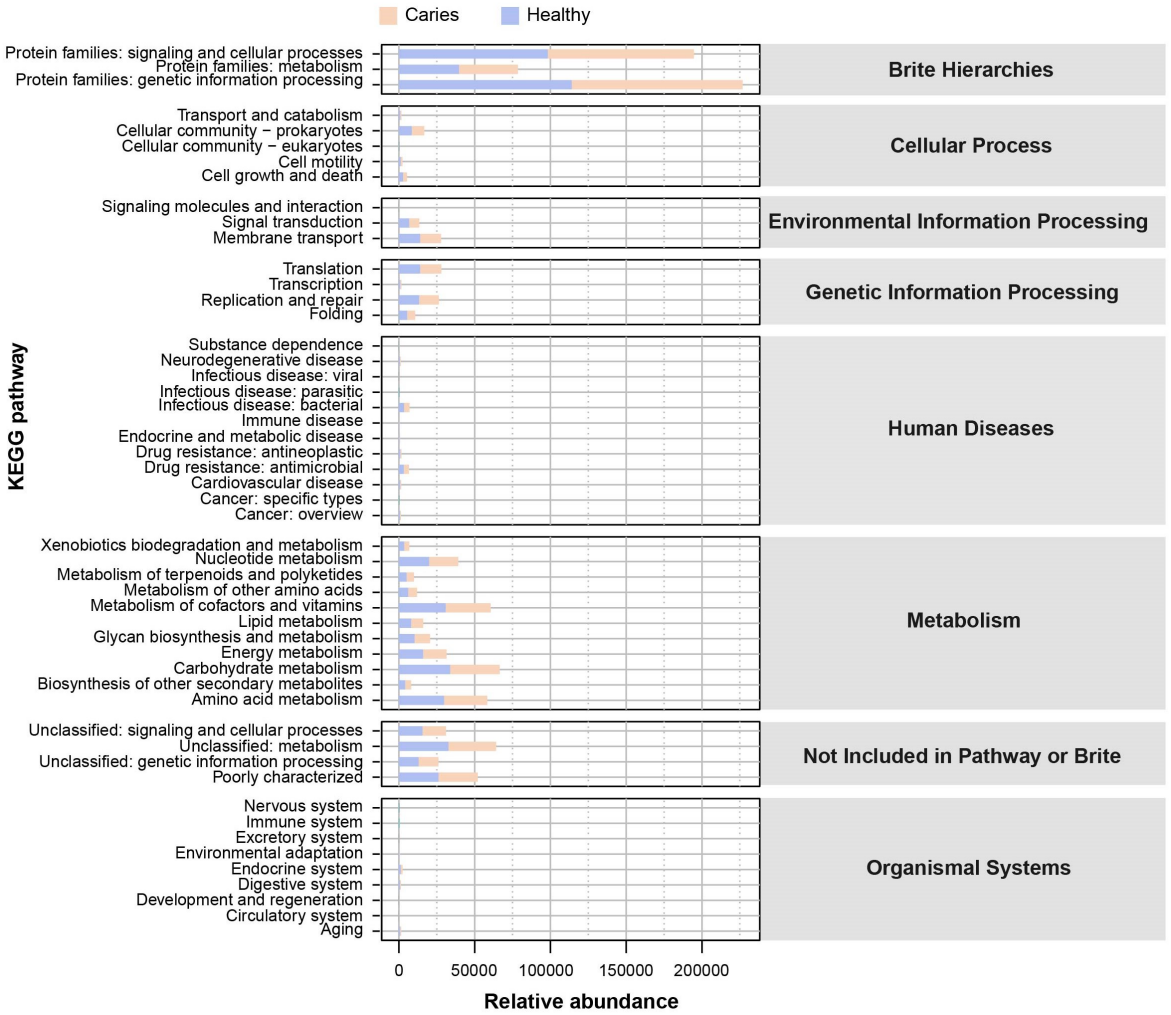
The outline functional analysis between S-ECC and caries-free children.

**Figure 6:**
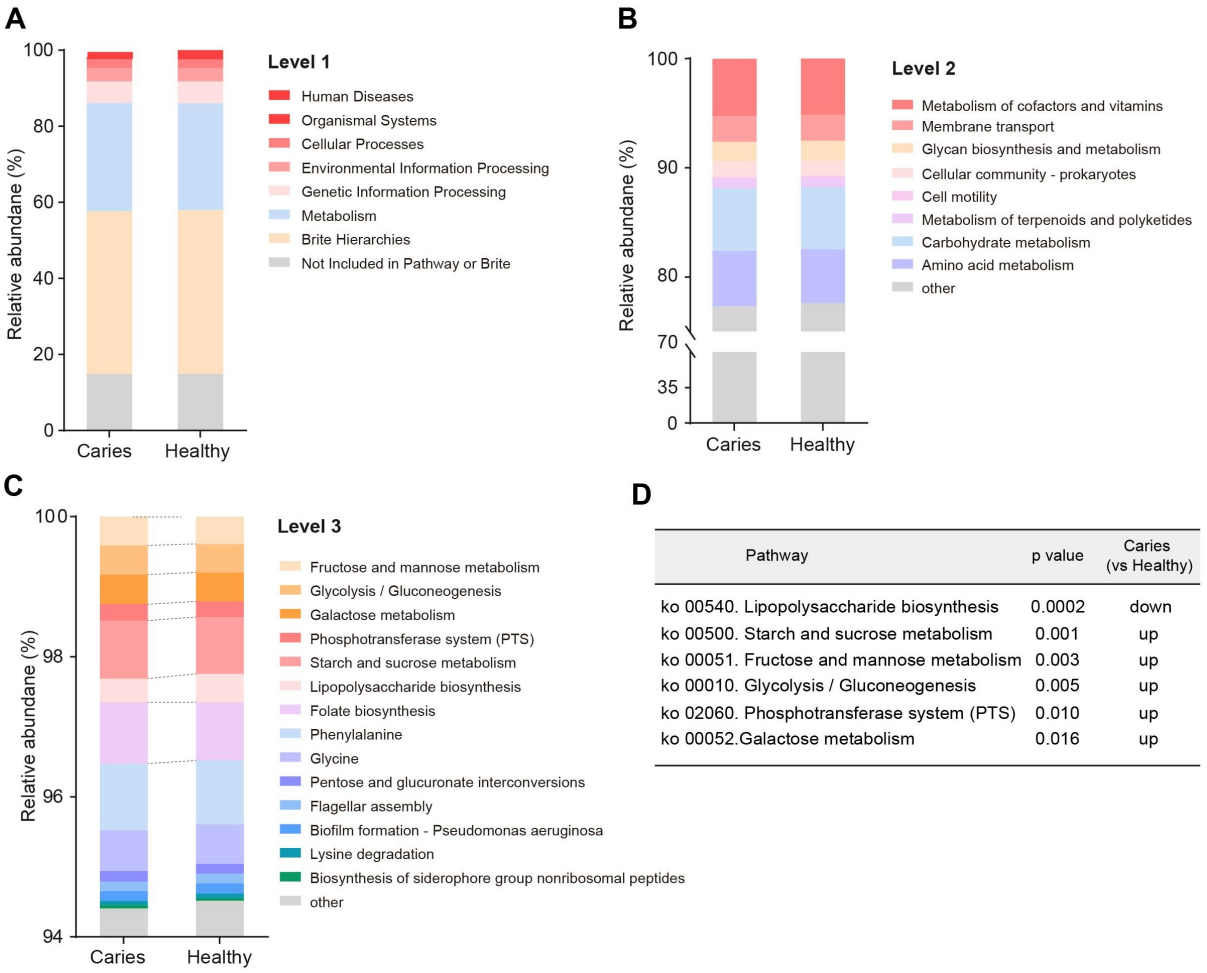
Functional profiles of salivary microbiome in children with and without caries. The comparison of functional pathways at the KEGG level 1 (A), level 2 (B), and level 3 (C). (D) The significantly altered functional pathways between two groups.

Since the microbial composition of saliva samples was distinct between caries and healthy groups, we tried to evaluate the functional profiles of oral microorganisms. By assembling genes, we obtained 2,420,045 genes in total and annotated the gene catalogues using KEGG (Kyoto Encyclopedia of Genes and Genomes) databases. Figure 5 describes the functional overview of two groups. We found that the great majority of oral microbial functions belonged to categories including Metabolism, Genetic information processing and Environmental information processing and Brite hierarchies, besides those not included in Pathway or Brite, whilst functional differences could not be provided in this figure pattern. To get a systematic understanding, we conducted KEGG analysis at levels 1/2/3 to display the function pathways. At level 1 and level 2, there was no obvious difference between children with and without caries (Figs. 6A and 6B). When down to level 3, several pathways involving carbohydrate metabolism were differentially regulated (Fig. 6C). According to the enrichment analysis of metabolic pathways, starch and sucrose metabolism (ko00500), fructose and mannose metabolism (ko00051), glycolysis/gluconeogenesis (ko00010), phosphotransferase system (PTS, ko02060), galactose metabolism (ko00052), pentose and gluconate conversions (ko00040), and folate biosynthesis (ko00790) were significantly up-regulated in S-ECC group (Fig. 6D), while lipopolysaccharide biosynthesis (ko00540) and biofilm formation-pseudomonas aeruginosa (ko02025) pathways went up in oral health children. Alterations of these functional pathways primarily reflected the changed growth and metabolic abilities of oral microorganisms, and probably led to the formation of dental caries.

## Discussion

Early childhood caries (ECC) refers to tooth decay occurring in children under the age of six, and is a significant and common oral disease in preschool children. ECC not only decays children’s teeth and induces tooth loss but also may lay psychological burdens on children, so it is of particular importance to take preventive interventions and therapeutic treatments targeting ECC. In light of the contributing roles of bacteria in caries formation, high-throughput sequencing and analytical methods in the flourishing microbiome studies have been introduced by researchers to investigate the crosstalk between oral flora and the occurrence of caries in children. In the study, we conducted shotgun sequencing to systematically analyze the salivary microorganisms in preschool children with and without severe caries from the aspects of microbial structure, composition, to functions. The microbial composition at the genus/species level was altered in caries children, as well as several carbohydrate metabolism-related pathways.

As for the regulation of caries on oral microbial diversity (*e.g.*, α-diversity and PCoA analysis), in the present study, we observed no significant differences in such parameters of the salivary microbiome between S-ECC children and caries-free children, highly consistent with the previous works (Yang et al., 2012; Jiang et al., 2016; Hurley et al., 2019). We speculate that the influence caused by primary dental caries maybe not enough to disturb the microbial structure. Of note, microbiota from saliva, plaque and cavity usually represent the oral microbiome in this field, thus different samples could generate distinct readouts of microbiome data. Hurley and colleagues once identified differences between the caries microbiota and saliva microbiota (Hurley et al., 2019), other groups also showed that microbial communities varied even at different parts of the oral cavity (Dewhirst et al., 2010; Aas et al., 2005). Therefore, researchers should bear it in mind to obtain a relative authentic analysis in future work.

The identification of representative genus/species to discriminate caries and healthy group is a meaningful finding in the study. For instance, we found that *Propionibacterium* and *Propionibacterium acidifaciens* were exclusively present in the saliva microbiota of S-ECC children, with prominent significance. This observation was rarely reported in previous studies. As a propionic acid-producing bacterium, *Propionibacterium* was once isolated from carious dentin and could decompose collagen and cause dentin caries (Hoshino et al., 1984). Another team has recently detected *Propionibacterium* in the microbial community from the apical area of persistent apical periodontitis (Zhou & Wang, 2014). These findings suggested *Propionibacterium* may be a conditional pathogen of dental diseases. *Propionibacterium acidifaciens* was first isolated from human oral cavity in 2009, and could produce acetic acid, propionic acid and succinic acid (Downes & Wade, 2009). It has been reported that *Propionibacterium acidifaciens* mainly invaded dentin and was often detected in dentin caries (Gross et al., 2012; Kianoush et al., 2014; Lima et al., 2011). Until recently, Obata et al. (2019) have unraveled the underlying mechanism by which *Propionibacterium acidifaciens* could closely combine with collagen in dentin and produce acids in low pH environment, in turn, its strong acid resistance enables *Propionibacterium acidifaciens* to survive in dentin tissue, ultimately leading to the formation of dentin caries. Based on these, we proposed that *Propionibacterium/Propionibacterium acidifacien* might be used as the potential microbial markers for primary dental caries.

Besides, the relative abundance of *Streptococcus mutans*, *Prevotella denticola* and *Actinomyces sp. oral taxon 448/414* was significantly higher in the S-ECC group than caries-free children, indicating that these significantly altered species may act as cariogenic bacteria to participate in acid production metabolisms to trigger caries. Actually, *Streptococcus mutans* has been widely recognized as the dominant cariogenic bacterium, due to its prominent ability to generate acid and extracellular polysaccharides and resist acid (Loesche, 1986; Lang et al., 1987; Palmer et al., 2010). Various studies on children with dental caries have revealed the high amount of *Streptococcus mutans* in the oral microbiome, again corroborating the provoking effect of *Streptococcus mutans* on caries development (Jiang et al., 2016; Hurley et al., 2019). *Prevotella*, a gram-negative anaerobic bacterium, is an important pathogen to infect root canals (Kumar et al., 2003) and its cariogenic capacity has been demonstrated by mounting evidence (Hurley et al., 2019; Yang et al., 2014; Teng et al., 2015; Kressirer et al., 2018). Therefore, it was reasonable that *Prevotella denticola* (a species of *Prevotella*) was significantly enriched in S-ECC group in our work. Zhang et al. (2020a) and Zhang et al. (2020b) recently studied the saliva samples of S-ECC children and oral health children by real-time PCR-based quantification method. They also found much higher levels of *Streptococcus mutans* and *Prevotella denticola* in children with caries, providing strong support for our study. For another species *Actinomyces sp. oral taxon 448/414*, little literature is released to illustrate their cariogenic roles and the underpinning mechanism, though the genus *Actinomycetes* has been regarded as the possible pro-caries bacteria. Given that *Actinomycetes* is a common bacteria in children’s oral cavity and can produce lactic acid, participate in the formation of dental plaque (Cisar, Kolenbrander & McIntire, 1979), it is plausible that *Actinomyces sp. oral taxon 448/414*, species of *Actinomyces*, may contribute to the onset and development of primary dental caries following a similar mechanism. Jiang et al. (2016) reported an increase of *Actinomyces graevenitzii* in the salivary microbiota of caries children by applying 16S rRNA sequencing, while no increase but mild decrease of *Actinomyces graevenitzii* was observed in our study. The probable explanation is that 16S rRNA sequencing and shotgun sequencing has different consistency and accuracy, especially at the species level (Xu et al., 2021).

Another interesting finding is that there are significant differences in the function profiles of salivary microbiota between the S-ECC group and the caries-free group. According to the enrichment analysis, multiple pathways involving carbohydrate metabolisms were obviously up-regulated in the S-ECC group, including starch and sucrose metabolism, fructose and mannose metabolism, glycolysis/gluconeogenesis, phosphotransferase system (PTS) and galactose metabolism. Generally, plaque bacteria utilize carbohydrates as the nutrient source for survival by executing carbohydrate metabolisms (Gao, 2013). The anaerobic fermentation is critical for the development of dental diseases, including caries, thereby the glycolysis/gluconeogenesis metabolism-related pathways will be active in populations with a high risk of caries. Studies have demonstrated that the dominant caries-pathogenic bacteria, *Streptococcus mutans* can consume a large amount of sucrose for glycolysis to generate acids. To enter into cells for the subsequent reactions, sucrose needs to be transferred *via* phosphotransferase system (PTS) (Hiratsuka et al., 1998). Zeng et al. (2017) have identified up to 15 enzymes of PTS in the isolates of *Streptococcus mutans*, providing theoretical evidence for its strong capacity to catabolize sucrose. These enzymes can metabolize monosaccharides (including glucose, fructose, mannose and galactose), as well as disaccharides (including sucrose, maltose and lactose, *etc.*) (Ajdić et al., 2002; Moye, Zeng & Burne, 2014). As such, the up-regulation of carbohydrate metabolisms could reflect the higher cariogenic potential in caries children than healthy kids. Intriguingly, we found that the lipopolysaccharide (LPS) biosynthesis pathway went down in the S-ECC group. As a typical glycolipid component of the cell wall of gram-negative bacteria, LPS could effectively prevent the entry of numerous toxic substances and enable bacteria to survive in varied environments (Fang, Zhao & Zhu, 2011; Zhang et al., 2020a; Zhang et al., 2020b). Besides, the continuous exposure of the host surface to trace LPS could in turn motivate the protective mechanism of bacteria (Fang, Zhao & Zhu, 2011), which might explain the high level of lipopolysaccharide biosynthesis pathway in caries-free children. However, it remains unclear what factors would provoke the beneficial reactions.

## Conclusions

Using the shotgun sequencing, we found that the salivary microbial communities varied between three-year-old children with and without caries, characterized by the significant differences in the specific genus/species and functional levels. Our study not only describes a comparative analysis regarding the oral microbiota between S-ECC and healthy children, but also provides microbial understandings of the cariogenic mechanism, which might contribute to the exploration of novel diagnostic approaches and interventions targeting ECC.

## Supplemental Information

10.7717/peerj.13529/supp-1 Supplemental Information 1 Subject information

## Additional Information and Declarations

### Data Availability

The following information was supplied regarding data availability: All raw sequencing data are available at the National Center for Biotechnology Information (NCBI): PRJNA791464.
